# Alterations of Methionine Metabolism as Potential Targets for the Prevention and Therapy of Hepatocellular Carcinoma

**DOI:** 10.3390/medicina55060296

**Published:** 2019-06-21

**Authors:** Rosa M. Pascale, Graziella Peitta, Maria M. Simile, Francesco Feo

**Affiliations:** Department of Clinical, Surgery and Experimental Sciences, Division of Experimental Pathology and Oncology, University of Sassari, 07100 Sassari, Italy; graziella.85@live.it (G.P.); simile@uniss.it (M.M.S.); feo@uniss.it (F.F.)

**Keywords:** hepatocarcinogenesis, methionine metabolism, S-adenosylmethionine, signal transduction, mechanisms of action of SAM, methyladenosyltransferases

## Abstract

Several researchers have analyzed the alterations of the methionine cycle associated with liver disease to clarify the pathogenesis of human hepatocellular carcinoma (HCC) and improve the preventive and the therapeutic approaches to this tumor. Different alterations of the methionine cycle leading to a decrease of S-adenosylmethionine (SAM) occur in hepatitis, liver steatosis, liver cirrhosis, and HCC. The reproduction of these changes in MAT1A-KO mice, prone to develop hepatitis and HCC, demonstrates the pathogenetic role of *MAT1A* gene under-regulation associated with up-regulation of the *MAT2A* gene (MAT1A:MAT2A switch), encoding the SAM synthesizing enzymes, methyladenosyltransferase I/III (MATI/III) and methyladenosyltransferase II (MATII), respectively. This leads to a rise of MATII, inhibited by the reaction product, with a consequent decrease of SAM synthesis. Attempts to increase the SAM pool by injecting exogenous SAM have beneficial effects in experimental alcoholic and non-alcoholic steatohepatitis and hepatocarcinogenesis. Mechanisms involved in hepatocarcinogenesis inhibition by SAM include: (1) antioxidative effects due to inhibition of nitric oxide (NO•) production, a rise in reduced glutathione (GSH) synthesis, stabilization of the DNA repair protein Apurinic/Apyrimidinic Endonuclease 1 (APEX1); (2) inhibition of *c-myc, H-ras,* and *K-ras* expression, prevention of NF-kB activation, and induction of overexpression of the oncosuppressor *PP2A* gene; (3) an increase in expression of the ERK inhibitor DUSP1; (4) inhibition of PI3K/AKT expression and down-regulation of *C/EBPα* and *UCA1* gene transcripts; (5) blocking LKB1/AMPK activation; (6) DNA and protein methylation. Different clinical trials have documented curative effects of SAM in alcoholic liver disease. Furthermore, SAM enhances the IFN-α antiviral activity and protects against hepatic ischemia-reperfusion injury during hepatectomy in HCC patients with chronic hepatitis B virus (HBV) infection. However, although SAM prevents experimental tumors, it is not curative against already established experimental and human HCCs. The recent observation that the inhibition of MAT*2*A and *MAT2B* expression by miRNAs leads to a rise of endogenous SAM and strong inhibition of cancer cell growth could open new perspectives to the treatment of HCC.

## 1. Introduction

Hepatocellular carcinoma (HCC) is the fifth most common human cancer, particularly frequent in areas where the infections by hepatitis B and hepatitis C virus are endemic or food is contaminated by Aflatoxin B1, such as sub-Saharan Africa and far eastern Asia [1,2,3]. Nonetheless, HCC incidence is also rising in Europe and the United States due to augmented incidence of hepatitis C virus infection, non-alcoholic steatohepatitis, associations with obesity, metabolic syndrome, and type 2 diabetes mellitus [1,2,3,4].

HCC is a fatal disease with survival expectancy no longer than six months after the time of diagnosis. Partial liver resection or liver transplantation could be curative. Small HCC lesions detected by ultrasonography may be successfully cured by resection or radiofrequency ablation [5]. However, only a minority is responsive to these treatments [2,5,6]. Furthermore, therapies with pharmacological agents (i.e., Sorafenib alone or in combination with other signaling inhibitors), trans-arterial chemo-embolization or yttrium-90 microspheres, and percutaneous ethanol injection do not significantly improve the prognosis of patients with advanced disease [2,5,6]. This situation requires new efforts to identify therapies that, combined with traditional treatments, improve their effectiveness.

S-adenosylmethionine together with folate concurs with the metabolism of the one-carbon units [7]. Methionine, an essential amino acid, is required for normal development and cell growth. In mammals, its metabolism is involved in the methionine cycle and related pathways, such as the transsulfuration pathway, which allows the conversion of methionine to homocysteine, and the polyamine biosynthesis [8]. Liver lesions induced by different xenobiotic compounds, including preneoplastic and neoplastic liver lesions, are associated with profound modifications of the methionine metabolism, whose pathogenetic role has been well proved [8]. Thus, various attempts have been directed to the correction of some of the major metabolic alterations involved in liver disease. This review summarizes and discusses the main objectives reached in this field.

## 2. The Methionine Cycle and Related Pathways

**Figure 1:**
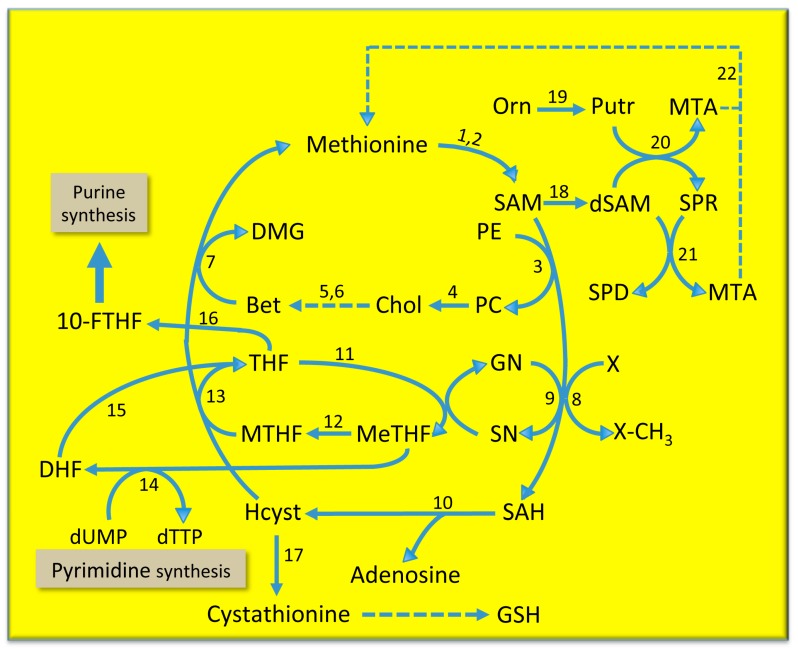
Metabolic cycles involved in methionine metabolism. *Substrates*: Bet, betaine; Chol, choline; DMG, dimethylglycine; dSAM, decarboxylated S-adenosylmethionine; GN, glycine; GSH, reduced glutathione; HCyst, homocysteine; Me-THF, 5,10-methylenetetrahydrofolate; MTA, 5-methylthioadenosine; MTHF, 5-methyltetrahydrofolate; MTR, methylthioribose; Orn, ornithine; PC, phosphatidylcholine; PE, phosphatidylethanolamine; Putr, putrescine; SAH, S-adenosylhomocysteine; SAM S-adenosylmethionine; SN, sarcosine; SPD, spermidine; SPR, spermine; THF, tetrahydrofolate. *Enzymes*: 1, MATI/III, methyladenosyltransferase I/III; 2, MATII, methyladenosyltransferase II; 3, phosphatidylethanolamine N–methyltransferase; 4, various phospholipases; 5, choline oxidase; 6, betaine aldehyde dehydrogenase; 7, betaine homocysteine methyltransferase; 8, glycine N-methyltransferase; 9, various methyltransferases; 10, S-adenosylhomocysteine hydroxylase; 11, methyltetrahydrofolate reductase; 12, 5-10-methylene-tetrahydrofolate reductase; 13, methionine synthetase; 14, thymidylate synthetase; 15, dihydrofolate reductase; 16, formyltetrahydrofolate synthetase; 17, cystathionine synthetase; 18, S-adenosylmethionine decarboxylase; 19, ornithine decarboxylase; 20, spermine synthetase; 21, spermidine synthetase; 22, 5-methylthioadenosine nucleosidase. The dotted arrow indicates the “salving pathway” for methionine resynthesis.

In mammals, methionine is involved in the so-called methionine cycle, the transsulfuration pathway, and the polyamine biosynthesis [9] (Figure 1). In the methionine cycle, methionine is converted to S-adenosylmethionine by methionine adenosyltransferases, methyladenosyltransferase I/III (MATI/III) and methyladenosyltransferase II (MATII). SAM is essential for the functioning of different metabolic pathways, such as various methylation reactions catalyzed by different methyltransferases or glycine methyltransferase that transforms glycine to sarcosine (methyl-glycine), the final product of which is S-adenosylhomocysteine (SAH).

SAH is a potent, competitive inhibitor of transmethylation reactions, thus its removal is necessary. S-adenosylhomocysteine hydroxylase transforms SAH to homocysteine. A specific synthetase transforms homocysteine to cystathionine that may be used for reduced glutathione biosynthesis. Alternatively, homocysteine may be used for methionine resynthesis. This resynthesis occurs by a reaction catalyzed by betaine homocysteine methyltransferase coupled with the pathway leading to betaine synthesis or with the folate cycle. Betaine synthesis follows the transformation of phosphatidylethanolamine to phosphatidylcholine by phosphatidylethanolamine methyltransferase (PEMT) in the so-called Bremer pathway (Figure 1) [10], in which different phosphatases transform phosphatidylcholine to choline, which is converted to betaine by reactions catalyzed by choline oxidase and betaine aldehyde dehydrogenase. The Bremer pathway is particularly active during choline deficiency [10]. The folate cycle may also provide methyl groups [11] the transformation of tetrahydrofolate (THF) to 5,10-methylenetetrahydrofolate (MeTHF) catalyzed by methyltetrahydrofolate reductase, which is coupled with the resynthesis of glycine from sarcosine. This is followed by the synthesis of 5-methyltetrahydrofolate (MTHF) catalyzed by 5,10-methylene-tetrahydrofolate reductase. MTHF is converted to THF by methionine synthetase, and the recovered methyl group is used to convert homocysteine to methionine (Figure 1). MeTHF can also be transformed to dihydrofolate (DHF) in a reaction catalyzed by thymidylate synthetase. Through this reaction, the folate cycle impacts deoxythymidyltriphosphate (dTTP) synthesis, while THF, after transformation to 10-formyl-THF by a specific synthetase, may be involved in purine synthesis [12,13,14,15].

The influence of the methionine cycle on cell growth also occurs through the polyamine synthesis (Figure 1). SAM and ornithine decarboxylated by specific decarboxylases produce decarboxylated SAM (dSAM) and putrescine, respectively, that are converted by spermine synthetase to spermine and 5-methylthioadenosine (MTA). Spermine plus dSAM are converted to spermidine and MTA. The latter is transformed by a specific nucleosidase to methylthioribose, which may be further utilized in the so-called “salvage pathway” to regenerate methionine [15].

## 3. Regulatory Mechanisms of the Methionine Cycle

**Figure 2:**
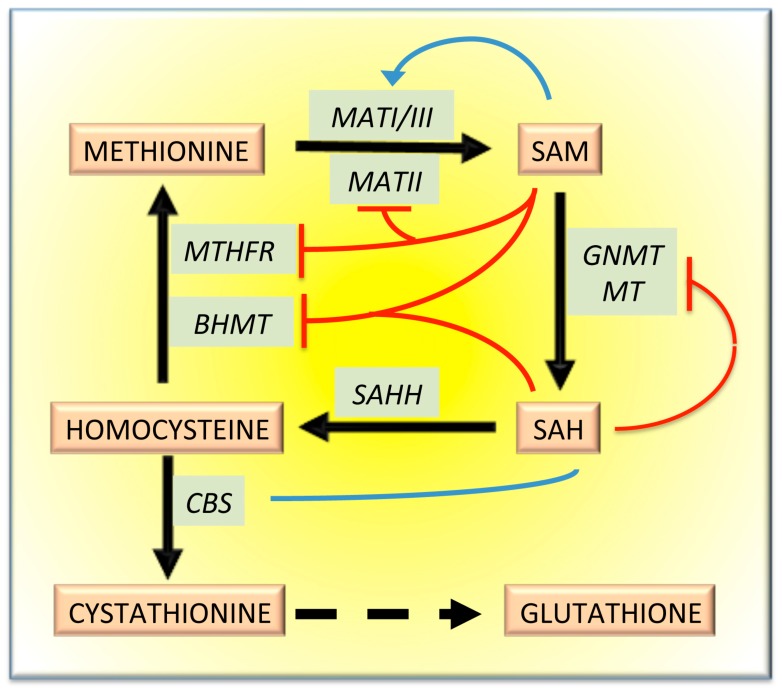
Regulation of the methionine cycle and GSH synthesis by SAM and SAH. *Abbreviations:* BHMT, betaine homocysteine methyltransferase; CBS, cystathionine synthetase; GNMT, glycine N-methyltransferase; MATI/III, methyladenosyltransferase I/III; MATII, methyladenosyltransferase II; MT, methyltransferase; MTHFR, methyltetrahydrofolate reductase; SAHH, S-adenosylhomocysteine hydroxylase. The blue arrows indicate activation; the red blunt arrows indicate inhibition.

The methionine cycle is under the control of several regulatory mechanisms. Various enzymes, including MATI/III, cystathionine synthetase (CBS), betaine homocysteine methyltransferase (BHMT) and glycine N-methyltransferase (GNMT), contribute to the maintenance of sufficient homocysteine concentrations. SAM and SAH “long-range” interactions [12] induce CBS activation (Figure 2). SAM decreases homocysteine re-methylation to methionine by allosterically inhibiting BHMT and methyltetrahydrofolate reductase (MTHFR), and the latter is also inhibited by SAH [16,17,18]. Thus, high SAM levels inhibit MTHFR and stimulate reduced glutathione (GSH) synthesis (Figure 2). Furthermore, methylation reactions are regulated by the SAH inhibitory effect on MT and GNMT [19]. Nevertheless, the reduction of the glycine remethylation could result in a certain decrease of the folate cycle. GNMT is controlled post-transcriptionally by phosphorylation [12] and is subjected to allosteric inhibition by MTHF [13,20,21,22].

SAM decarboxylation by a specific decarboxylase is a fundamental step of polyamine biosynthesis. MTA inhibits SAM decarboxylase [23]. This control step is regulated by the activity of MTA phosphorylase, which allows the methionine resynthesis in the “salvage pathway” (Figure 1).

A regulatory role of primary importance is played by the methyladenosyltransferases (MATs). *MAT1A* and *MAT2A* genes encode an α1 and an α2 subunit, respectively [24]. The α2 subunit, prevalently expressed in a fetal liver, is substituted by the α1 subunit in an adult liver. The MATI and the MATIII isozymes are, respectively, the tetramer and the dimer of the α1 subunit. The elevated Km of MATI/III for methionine indicates that the rise of methionine concentrations results in pronounced SAM synthesis. The SAM/SAH ratio plays a key role in SAM-dependent methyltransferase reactions [24] (Figure 2). The Km for methionine of MATI and MATIII is 23 μM–1 mM and 215 μM–7 mM, respectively. Therefore, SAM, at physiological liver level (about 60 μM), slowly inhibits MATI and stimulates MATIII activity (Figure 2). In contrast, the lower Km for methionine of MATII (4–10 μM) causes its inhibition by the reaction product (Figure 2) [24]. A third gene, *MAT2B*, encodes the MATs β-subunit without catalytic activity, which regulates MATII activity by lowering its Km for methionine and its inhibition constant (Ki) for SAM [24]. The β-subunit associates with the α-subunit; this association raises MATII susceptibility to the inhibition by SAM [25].

The mechanisms regulating *MAT2B* expression are poorly known. Two dominant splicing variants of *MAT2B*, V1 and V2, are upregulated in HCC [23]. TNF-α upregulates and SAM inhibits *MAT2B* V1 promoter expression through mechanisms involving ERK and AKT signaling [25]. Different proteins are regulated by MAT2B through physical interaction [23,24,25,26,27]. Among them, the G-protein-coupled receptor kinase-interacting protein 1 and the MEK1/2–ERK1/2 signaling pathway (promoting colon cancer cell proliferation) are activated by MAT2B [28].

**Figure 3:**
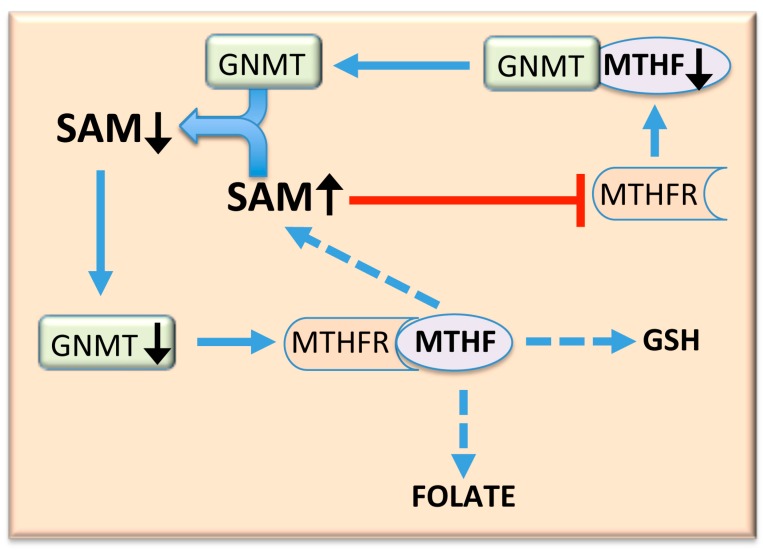
The GNMT salvage pathway. GNMT binds to and may be inhibited by MTHF. Therefore, when SAM levels increase, MeTHFR inhibition leads to a decrease in free MTHF and dissociation of the complex GNMT–MTHF. The consequent rise in free GNMT prevents the increase of SAM level. The decrease of SAM concentration is associated with a fall in free GNMT and with a release of MTHFR inhibition, leading to a rise in MTHF availability. The latter may be used for SAM resynthesis, GSH synthesis, and a rise in folate content. The blue arrows indicate activation; the red blunt arrows indicate inhibition; the black arrows indicate increase/decrease.

The SAM/SAH ratio and the cellular methylation reactions are strongly regulated by GNMT [29]. The latter provides an alternative way for conversion of SAM excess to SAH (Figure 1). Low SAM levels favor homocysteine remethylation, whereas high SAM levels activate CBS (Figure 2). Furthermore, GNMT is abundant in the liver; its product, sarcosine, which has no known physiological role, is converted back to glycine by sarcosine dehydrogenase (methyltetrahydrofolate reductase; Figure 1). GNMT is a major regulator of the cellular SAM/SAH ratio and SAM-dependent methyl transfer reactions. SAM-dependent methyltransferases are generally strongly inhibited by the product SAH, and the cellular SAM/SAH ratio plays a key role in methyl-transfer reactions [30]. Unlike most SAM-dependent methyltransferases, GNMT has a relatively high Km value for SAM and is weakly inhibited by SAH—the Ki value of GNMT for SAH is 35–80 μM, much higher than that of other methyltransferases [14]. Therefore, at physiological levels of SAM (76 nmol/g of rat liver and 46.21 nmol/g of mouse liver) and SAH (35.1 nmol/g of rat liver and 29.73 nmol/g of mouse liver) [31,32], GNMT exhibits appreciable activity. The fluctuations in GNMT activity could alter the SAM/SAH ratio, thus influencing the activity of methyltransferases. Furthermore, GNMT, as a major hepatic folate binding protein, binds to and may be inhibited by MTHF [27,28,29,30]. Therefore, when SAM levels increase, MeTHFR inhibition leads to a decrease in free MTHF and a dissociation of the complex GNMT-MTHF (Figure 3) [13,33,34]. The consequent rise in free GNMT prevents the SAM level from rising excessively (Figure 3). Conversely, when SAM concentration tends to decrease, the amount of free GNMT falls, MeTHFR inhibition is released, and more MTHF is available. Thus, GNMT increases cells’ folate content and the remethylation of MTHFR-dependent homocysteine. For this reason, the GNMT pathway may be considered a “salvage pathway” (Figure 3). Interestingly, various polymorphisms and loss of heterogeneity have been found in human GNMT [35].

Most studies on the methionine cycle focus on cell cytoplasm, where the role of the key enzymes of this pathway has been prevalently evaluated. Recent mounting evidence indicates that methionine adenosyltransferases (MATs), S-adenosylhomocysteine hydrolase (AHCY), GNMT, and BHMT [36,37,38,39,40,41] are also localized in the nucleus. It is known that the methyltransferases locations vary according to their functions [42]. However, the relationships between the subcellular localization of these enzymes and their alteration in hepatocarcinogenesis has not yet been clarified to date.

## 4. Alterations of the Methionine Cycle during Liver Injury

A link between the alterations of the methionine cycle and liver injury was, for the first time, suggested by the original Copeland and Salmon’s experiments showing the development of HCC in rats chronically fed a choline-deficient diet [43]. This pioneering observation, although difficult to interpret because of the possible presence of Aflatoxin B1 and other carcinogenic contaminants in the diet, focused the interest on the role of lipotropic deficiency in carcinogenesis. This role was confirmed by the observation that ethionine, an antagonist of the methyl donor aminoacid methionine, causes cancer [44] Further experiments from different laboratories confirmed that prolonged feeding of uncontaminated diets devoid of choline and methionine induces HCC development [45,46,47] preceded by lipid peroxidation [48], liver steatosis, and cirrhosis [49]. Feeding rats with a methyl-deficient diet induces a fall in SAM liver content [50]. A decrease in SAM content has also been demonstrated in preneoplastic and neoplastic liver induced by different treatments in rats fed adequate diets [51,52,53,54], as well as in human HCC [55].

Further studies demonstrated the down-regulation of the key enzymes of the methionine cycle, including MAT1A, GNMT, BHMT, CBS, and methionine synthetase (MS) in human cirrhosis and HCC [56], which may explain the hypermethioninemia, the hyperhomocysteinemia, and the decreased hepatic GSH levels observed in cirrhosis.

Experiments aimed at elucidating the mechanisms of the SAM fall in preneoplastic and neoplastic livers showed a decrease in *MAT1A* expression with concomitant *MAT2A* up-regulation in cirrhotic and neoplastic livers of rodents and humans, resulting in a decrease of the MAT1A:MAT2A ratio (MAT1A/MAT2A switch) [56,57,58]. The overexpression of the MATII enzyme does not compensate for the decrease in MATI/III isozymes because of MATII inhibition by its reaction product [57]. Thus, the fall in the MATI/III:MATII activity ratio associated with a rise in SAM decarboxylation and polyamine synthesis [59] induces a sharp decrease in SAM level.

The fundamental role of MAT1A downregulation in the pathogenesis of liver injury and HCC was further proved by the demonstration that, in MAT1A-KO mice, chronic SAM deficiency not compensated by MAT2A induction provokes precocious hepatomegaly with macrovesicular steatosis, involving up to 75% of hepatocytes, followed by mononuclear cell infiltration in periportal areas and HCC at eight months of age [59]. These mice exhibit an increased susceptibility to injury, expressing markers of an acute phase response and displaying increased proliferation [60]. Interestingly, a rise in oxidative stress associated with genomic instability and under-expression of APEX1 (Apurinic/Apyrimidinic Endonuclease 1) [61], a protein involved in DNA base excision repair [62], occurs in MAT1A-KO mice. In these mice, a decreased expression of the ERK inhibitor, DUSP1, associated with a rise in its proteasomal degradation, has been demonstrated [63]. These findings emphasize the role of the decrease in the MAT1A:MAT2A ratio and the SAM level in the onset and the progression of HCC.

Different mechanisms are involved in *MAT1A* downregulation in HCC. They include the methylation of the *MAT1A* promoter and the coding region at +10 or +88 positions, histone H4 deacetylation, and, at a post-transcriptional level, the interaction of MAT1A mRNA with the AUF1 protein that increases its decay [64,65,66,67,68,69]. Furthermore, miR-664, miR-485-3p, and miR-495 overexpressed in HCC were shown to inhibit MAT1A at the mRNA level [68]. *MAT2A* gene upregulation in HCC largely depends on hypomethylation of its promoter and histone H4 acetylation, while MAT2A mRNA stability is raised by interactions with the HuR protein [64,65,66]. Furthermore, Sp1, c-Myb (avian myeloblastosis viral oncogene homolog), NFkB (nuclear factor kappa B), and AP-1 transactivate MAT2A in HCC [69].

## 5. The Effects of Variations of Cellular SAM Pool

The effects of variations of SAM liver content have been evaluated in different pathologic conditions associated with a decrease of the cellular SAM pool. SAM administration to rats antagonizes liver damage induced by either galactosamine [70] or acetaminophen [71]. It also prevents the steatosis in ethanol-intoxicated rats and mice, an effect that was largely associated with the SAM role in maintaining an adequate reduced glutathione liver content [72,73,74]. These SAM effects were confirmed in ethanol-intoxicated baboons [75]. SAM was also found to normalize the ethanol-induced impairment of the transport of GSH into mitochondria mediated by a decreased fluidity of the mitochondrial inner membrane [76]. It also prevents TNFa-mediated glutathione depletion, ameliorates steatosis, hepatocyte necrosis, and elevates alanine aminotransferase (ALT) in ethanol intoxicated mice [76]. Interestingly, lipid peroxidation and fibrogenesis induced in rat liver by carbon-tetrachloride intoxication were also prevented by the antioxidant properties of MTA [77]. Some clinical trials have suggested that SAM effectively reduces steatosis in the liver of human suffering of chronic liver disease with a history of ethanol intake [78,79,80,81,82,83] and increases GSH liver content [75,76].

The decrease in SAM liver content in ethanol intoxicated rats, which is associated with a fall of phosphatidylethanolamine methyltransferase (Figure 1), results in a decreased methylation of phosphatidylethanolamine (Bremer pathway) [84] that is restored by SAM treatment [84,85]. A decrease in liver SAM content, which is associated with a phosphatidylcholine depletion, is induced in non-alcoholic steatohepatitis (NASH) by the oxidative stress resulting from free radicals generated by cytochrome P4502E1 (CYP2E1) [86]. A therapeutic treatment with SAM plus dilinoleoylphosphatidylcholine was found to be effective in the treatment of rats with experimentally induced NASH [86].

A decrease in SAM content and SAM/SAH ratio also occurs in rat liver during the development of preneoplastic liver foci induced by different experimental models and is still present in dysplastic nodules (DN) and HCC several weeks after the interruption of carcinogen treatments [53,55,68,87,88,89,90]. A fall in SAM content also occurs in human HCC [65], where the SAM level is inversely correlated with the degree of HCC progression, it being minimal in HCC with poorer prognosis [65]. SAM content decreases to a lower extent in the surrounding cirrhotic liver [65]. Kaplan–Meier survival curves of human HCC with high and low MATI/III:MATII ratios showed about a three-fold shorter survival in patients with lower MATI/III:MATII ratios [65]. Interestingly, the SAM content of HCC seems to be under genetic control, it being very low in rapidly progressing HCCs induced in F344 rats genetically susceptible to hepatocarcinogenesis, whereas little SAM decrease occurs in slowly growing tumors induced in the genetically resistant Brown Norway (BN) rats [55].

The treatment of rats with SAM during HCC induction by different carcinogens and hepatocarcinogenesis protocols strongly prevents tumor development [53,55,68,88,89,90]. Furthermore, forced expression of *MAT1A* in human HCC cells was found to suppress *in vivo* tumorigenicity in mice [91] and Huh7 cell transfectants, stably overexpressing *MAT1A*, and showing higher SAM levels, lower HCC growth rates, microvessel density, CD31 and Ki-67 staining, and higher apoptosis with respect to control tumors [91]. These findings robustly suggest a chemopreventive effect of SAM, which has been confirmed in an orthotropic HCC model, where SAM inhibited HCC development induced by the injection of H4IIE human HCC cells in the rat liver parenchyma [92]. However, the intravenous infusion of SAM for 24 days did not affect the size of already established tumors, probably due to the prevention of SAM accumulation by the compensatory induction of hepatic GNMT [92]. These findings confirm the chemopreventive effect of SAM and demonstrate that SAM has no curative effect—at least in the adopted experimental conditions. It is important to note, in this respect, that GNMT is often silenced in more aggressive rat and human HCCs [93]. Therefore, the question of whether SAM has a curative effect against these tumors still requires a definitive response.

Interestingly, SAM and MTA were found to prevent experimental colon carcinogenesis [94]. Both compounds reduce chronic inflammation, which represents a main risk factor for this type of tumor [94]. In vitro growing human colon cancer cells exhibit *MAT2A* overexpression, whose silencing induces apoptosis [94].

## 6. The Mechanism of the S-Adenosylmethionine Antitumor Effect

Early research on the antitumor action of SAM focused on the inhibition of polyamine synthesis. Ornithine decarboxylase overexpression and overactivity characterizes hepatocarcinogenesis [51,95,96]. The treatment of rats with SAM during early stages of hepatocarcinogenesis strongly inhibits Ornithine decarboxylase (ODC) and polyamine synthesis in preneoplastic liver lesions [95,96]. The mechanism of the inhibitory action of SAM on ODC activity has not been definitively clarified. ODC inhibition could be attributed to the accumulation of MTA, an end-product of polyamine synthesis (Figure 1), as well as a product of spontaneous splitting of SAM at a physiological pH and temperature [97]. However, no MTA accumulation occurs in preneoplastic and neoplastic liver lesions, probably due to MTA utilization for methionine resynthesis through the salvage pathway [15].

Further studies from different laboratories have shown that multiple mechanisms concur with the inhibition of hepatocarcinogenesis by SAM.

### 6.1. SAM and MTA Contribution to Genomic Stability

It is widely accepted [98,99,100] that the interaction of DNA with carcinogens and reactive oxygen and nitrogen species generated during carcinogen metabolism and/or inflammation accompanying early stages of hepatocarcinogenesis results in genomic instability (GI), leading to somatic point mutations, copy number alterations of individual genes, and gain/loss of chromosomal arms.

**Figure 4:**
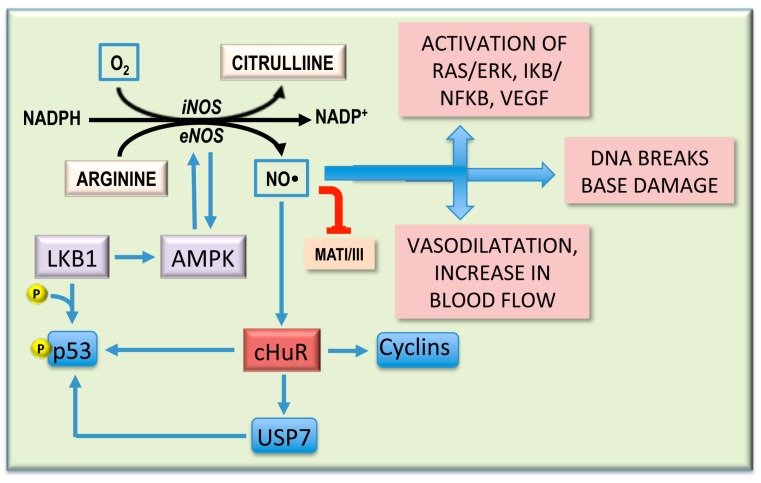
Synthesis and effects of nitric oxide (NO•). NO• induces the nuclear to cytoplasmic HuR translocation, resulting in stabilization of *cyclins, p53,* and *USP7* mRNAs. Hyperactive LKB1 induces p53 hyperphosphorylation. The interaction of phosphorylated p53 with USP7 blocks the negative regulation of p53 by MDM2. LKB1 also activates AMPK that induces endothelial NOS (eNOS) and is induced by the latter. The blue arrows indicate activation; the red blunt arrows indicate inhibition.

Nitric oxide (NO•) is a product of L-arginine to L-citrulline conversion by nitroxide synthetase (NOS; Figure 4). Hepatocytes, cholangiocytes, and Kupffer and stellate cells contain a calcium-independent, inducible NOS (iNOS), while a calcium-dependent endothelial NOS (eNOS) is present in endothelial cells [101]. NO• production by iNOS activation during chronic hepatitis may favor hepatocarcinogenesis by inducing DNA mutations and vasodilation, providing premalignant and malignant cells with sufficient metabolites and oxygen [101,102] (Figure 4). Inhibition of iNOS by aminoguanidine causes NF-kB and RAS/ERK downregulation, decreases HCC cell growth, and enhances apoptosis in vivo and in vitro [102]. eNOS is activated by AMPK during hepatocarcinogenesis [103]. NO• production may further activate AMPK [102], thus establishing a vicious circle (Figure 4). The role of the LKB1/AMPK axis in hepatocarcinogenesis is also supported by the observation of LKB1/AMPK activation in MAT1A-KO mice and MATI/III inactivation by NO• [103] (Figure 4). LKB1/AMPK activation is also induced by hepatocyte growth factor (HGF) and blocked by SAM [104].

The SAM antioxidative action may also be attributed to its support to the GSH pool. Indeed, SAM treatment maintains a high pool of reduced glutathione in CCl_4_-intoxicated rats [105]. The DNA protection from oxidative damage by antioxidants is known to prevent tumor development in livers and other tissues [105,106,107,108]. Additionally, MTA was found to exert an antioxidative effect [77], which has been attributed to sulfoxide and sulfone derivatives of MTA oxidation by microsomal mono-oxygenases [109]. Interestingly, acidreductone dioxygenase 1 (ADI1), a tumor suppressor often under-regulated in HCC, was found to increase SAM levels by promoting the MTA cycle (SAM salvage pathway), resulting in a higher availability of methionine for SAM synthesis [110]. However, SAM may exert an antitumor action independently of MTA. In fact, in vitro growing stable transfectants of the liver tumor Huh7 cell overexpressing *MAT1A* exhibit higher SAM levels and no change in MTA content and are less tumorigenic in vivo than non-transfected Huh7 cells [111].

A defense against GI is operated by the DNA repair protein, APEX1, an apurinic endonuclease stimulated by reactive oxygen species (ROS) [112,113,114]. The APEX1 mRNA and protein expression exhibit 20% and 50% decreases, respectively, in MAT1-KO mice, associated with a rise in AP sites and downregulation of APEX1 targets, including Bax, Fas, and p21 [111]. The decrease in *MAT1A* mRNA in in vitro growing human and mouse liver cells is associated with a 60% fall of APEX1 protein, which is prevented by SAM [64]. This suggests that APEX stabilization contributes to the SAM chemopreventive action. The mechanism of APEX1 stabilization by SAM is not completely known. The ubiquitin-conjugating enzyme 9 (Ubc9) induces the proteasomal degradation of the redox factor-1 (Ref-1) involved in cellular redox regulation and DNA AP site repair [115]. It has been found that SAM inhibits the chymotrypsin-like and the caspase-like activities of the 26S proteasome as well as the expression of cell division cycle 2 (CDC2) elevated in different tumors, with a consequent decrease of Ubc9 phosphorylation and proteasomal degradation [116].

### 6.2. SAM and Signal Transduction

**Figure 5:**
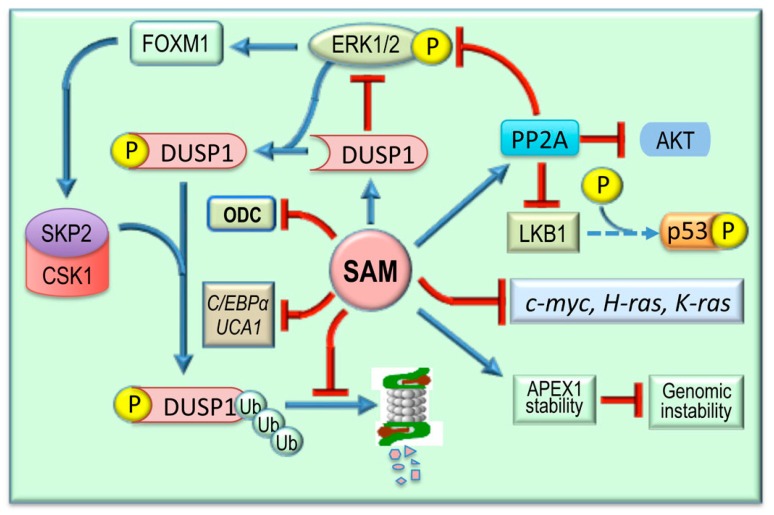
Effects of SAM on signal transduction pathways. SAM is involved in the stabilization of the DNA repair enzyme APEX1, thus reducing genomic instability. Through the inhibition of LKB1/AMPK axis, SAM controls p53 phosphorylation and cell growth and survival by inducing PPA2 expression that phosphorylates and inactivates AKT and its targets. Moreover, PPA2 activation and DUSP1 stabilization inhibit the RAS/ERK pathway. The inhibition of ERK1/2 activity by DUSP1 is controlled by DUSP1 phosphorylation of Ser296, which allows its ubiquitination by the SKP2–CSK1 ubiquitin ligase and proteasomal degradation, as well as by SKP2–CSK1 activation operated by FOXM1, a major target of ERK1/2. SAM also affects the cell cycle by inhibiting *c-MYC, H-ras,* and *K-ras* expression and ODC activity. Finally, the antitumor effects of SAM could also be exerted through inhibition of *C/EBPα* and *UCA1* expression.

The inhibition of the growth of preneoplastic liver lesions by SAM suggests an inhibitory effect of the latter on the signal transduction pathways supporting the fast growth of tumor cells. Early observations on the influence of SAM on these pathways show that the treatment of rats with SAM during the development of preneoplastic liver lesions inhibits the expression of *c-myc, H-ras,* and *K-ras* [87] (Figure 5) as well as ODC activity [51]. This, at least for *c-myc. H-ras,* and *K-ras*, was shown to depend on the reversion of the hypomethylation status of these genes [87,117]. Further studies have demonstrated that the inhibition of various signaling pathways is involved in the SAM antitumor effect. SAM down-regulates Dead-box protein 3 (DDX3X), a RNA helicase regulating RNA splicing, export, transcription and translation [118]. Moreover, the treatment of rats with SAM during the development of preneoplastic foci induces the overexpression of the oncosuppressor *PP2A* gene, which regulates a great portion of the phosphoproteome, including pathways involved in apoptosis, proliferation, and DNA damage response [119,120] (Figure 5). 

SAM also provokes a decrease of *ERK1/2* activity by interfering with the ERK inhibitor, DUSP1. ERK1/2 upregulation is associated with low DUSP1 expression in fast growing DN and HCC of F344 rats genetically susceptible to hepatocarcinogenesis and in human HCCs with poor prognosis [121]. This may partially depend on the phosphorylation by ERK1/2 of DUSP1 Ser296, followed by DUSP1 ubiquitination by the SKP2-CKS1 ubiquitin ligase and proteasomal degradation [121,122] (Figure 5). ERK1/2 sustains SKP2-CKS1 activity via its target FOXM1, which mediates the ERK1/2 effects on cell cycle, cell survival, and angiogenesis [123] (Figure 5). In accordance with these findings, Dusp1 mRNA and proteins are highly decreased in the livers of MAT1A-KO mice and in cultured mouse and human hepatocytes [63]. SAM administration to these mice induces a rise in Dusp1 mRNA and protein expression and a decrease in ERK activity [63]. Furthermore, SAM prevents a DUSP1 mRNA and protein fall in in vitro cultured human and mouse hepatocytes, probably by inhibiting its proteasomal degradation [63]. Interestingly, forced expression of *MAT1A* in human hepatoma cells suppresses in vivo tumorigenicity in mice [93]. FOXM1 expression is also sustained by the TNF-α/HIF-1α axis [124]. The hypoxia may reduce the SAM level of HCC cells through HIF-1α binding to the MAT2A promoter [125].

Further antitumor effects of SAM seem to be exerted through the inhibition of the long noncoding RNAs, C/EBPα and Urothelial cancer associated 1 (UCA1) [126]. Recent observations showed an elevation of C/EBPα dephosphorylated at Ser190/193 in the Pten/p53 double knockout mice model and in a large cohort of human hepatoblastomas [127]. Furthermore, dephosphorylated C/EBPα induced preneoplastic foci containing cancer stem cells that evolved into HCCs and aggressive hepatoblastomas, isolated C/EBPα-dependent multinucleated hepatocytes, and exhibited elevation of stem cell markers [127]. C/EBPα-dependent cancer stem cells were found in patients with aggressive hepatoblastomas and in patients predisposed to liver cancer [127]. UCA1 is another gene upregulated in HCC tissues and cell lines whose expression is associated with malignant behavior [128]. Evidence was found indicating that UCA1 plays a crucial role in HCC proliferation through the Hippo signaling pathway [128]. SAM given to rats at the starting phase of diethylnitrosamine-induced hepatocarcinogenesis was found to down-regulate C/EBPα and UCA1 gene transcripts and reduce the histopathological alterations in HCC [126]. Interestingly, it was observed that the inhibitory effects of SAM on *C/EBPα* and *UCA*1 genes were mediated by the inhibition of PI3K/Akt protein expression [126].

An important contribution to hepatocarcinogenesis is given by the activation of LKB1/AMPK. LKB1 activates AKT independently of PI3K, AMPK, and mTORC [127]. The activation of hepatocyte AMPK provokes nuclear to cytoplasmic translocation of HuR with consequent stabilization of Cyclins mRNA and cell proliferation [128] (Figure 4). The hyperphosphorylation and the cytoplasmic retention of p53 by different kinases, including LKB1, allows its interaction with the de-ubiquitinating enzyme, USP7, with consequent blocking of the negative regulation of p53 by MDM2 [129]. Furthermore, cytosolic HuR stabilizes p53 and USP7 mRNAs [128] (Figure 5). SAM blocks LKB1/AMPK activation [128]. Notably, cytoplasmic staining of p53 and p-LKB1 (Ser428) occurs in NASH and HCC of MAT1A-KO mice and in liver biopsies of human HCC induced by ASH and NASH [128]. However, these observations contrast with the LKB1 loss found in different tumors, including HCC [130]. *LKB1* is considered an oncosuppressor gene [131], and AMPK activated by LKB1 inhibits AKT signaling by triggering the TSC2/TSC1 oncosuppressor complex [132]. It must also be considered that the downregulation of the AMPKa2 catalytic subunit is present in undifferentiated HCC, and AMPK inactivation promotes hepatocarcinogenesis by destabilizing p53 in a p53 deacetylase- and a SIRT1-dependent manner [132].

SAM may also protect the JAK/STAT signaling in Hepatitis C virus (HCV)-induced liver damage. The HCV protein damages JAK-STAT signaling by the inhibition of STAT1 methylation, resulting in STAT1 binding to its inhibitor, PIAS1 [133]. SAM and betaine restore STAT1 methylation and improve the IFNα antiviral effect in in vitro growing cells [133].

### 6.3. SAM and the Warburg Effect

It is well known that the high glucose consumption by cancer cells is associated with a restraint of oxygen consumption and lactic acid production in aerobiosis [134,135,136]. This does not seem to depend on functional alterations of tumor mitochondria. The mitochondria isolated from different liver tumors were found to be well-coupled and responsive to functional stimuli, such as changes of substrates and adenosine diphosphate (ADP) concentration [137]. The respiratory impairment of tumors in aerobiosis largely depends on the use of high ADP amounts for the production of glycolytic ATP, while the decreased availability of mitochondrial ADP limits oxygen consumption [136]. Warburg hypothesized that the glycolytic metabolism of cancer cells (also known as the “Warburg effect”) was involved in carcinogenesis [134]. Accordingly, the inhibition of glycolysis by a hexokinase inhibitor (2-deoxyglucose) in the rat hepatocarcinoma ascites AH-130, characterized by high lactic acid production in aerobiosis, led to a strong inhibition of protein synthesis, while it was without effect in normal cells [138]. Furthermore, recent research showed that different inhibitors of the glycolytic pathway, including 2-deoxyglucose, inhibit YAP/TAZ signaling, which is active in mammary and liver tumors, i.e., in cells with active glycolytic metabolism [139]. It was also demonstrated that the glycolytic enzyme PFK-1 (phosphofructokinase 1) binds the cofactors TEADs, promoting their cooperation with YAP/TAZ [139], a pathway implicated in the acquisition of stemness properties of HCC cells [140]. Furthermore, AMPK upregulation in cancer cells activates phosphofructokinase 2 (PFK-2), a key enzyme of glycolysis [141], and CHIP (carboxyl terminus of Hsc70-interacting protein), a U-box E3 ligase, inhibits the aerobic glycolysis and the progression of ovarian cancer by inhibiting PFK-2 [142].

The glycolytic metabolism of cancer cells is largely regulated by oncogenes. The hexokinase activity is enhanced by c-*MYC* and *AKT*, pyruvate kinase and lactate dehydrogenase are activated by c-*MYC* and *HIF-1α*, and glucose transport is activated by c-*MYC, HIF-1α,* and *AKT,* while *RAS* activates pyruvate kinase [143,144,145]. *HSF-1α* also activates glucose-6-phosphate dehydrogenase, an enzyme that provides pentose phosphates for nucleic acid synthesis [146,147]. Interestingly, *HSF-1α* and *MYC* contribute to maintain the low respiratory rate of cancer cells in the presence of glucose by activating the pyruvate dehydrogenase kinase that, by triggering pyruvate dehydrogenase activity, hampers the synthesis of acetyl-CoA [143].

**Figure 6:**
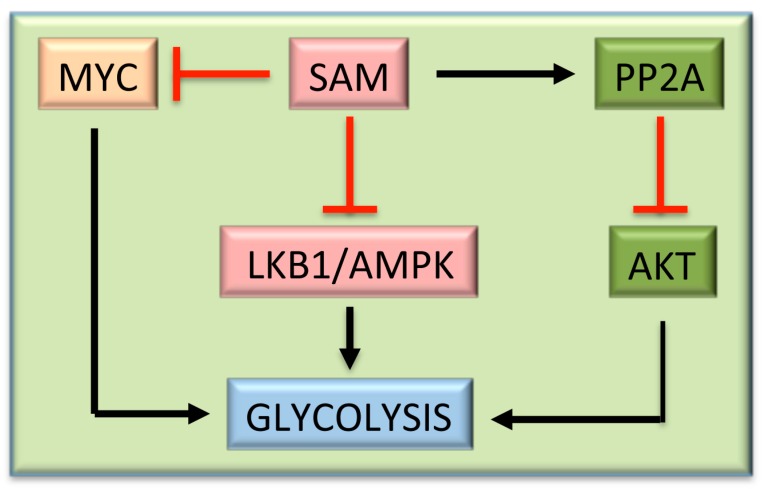
Effects of SAM on glycolysis. Black arrows indicate activation, blunt red arrows indicate inhibition.

It is interesting to note that many of the genes activating the glycolytic pathway are overexpressed in HCC and are sensitive to the SAM inhibitory effect [130]. Thus, the inhibition by SAM of c-*MYC* [87] and *LKB1/AMPK* [130] and the activation of the AKT inhibitor, PP2A [148], induce a restraint of glycolysis (Figure 6). Furthermore, during the development of preneoplastic foci, the glucose used for the synthesis of triacylglycerol and pyruvate decreases in rat livers, whereas a rise of reducing equivalents and pentose phosphates may favor DNA synthesis and detoxification reactions [149]. In SAM-treated rats, a partial reversion of carbohydrate metabolism to that present in normal livers associated with a decrease of DNA synthesis occurs [149].

### 6.4. DNA and Protein Methylation

SAM deficiency during hepatocarcinogenesis is associated with global and gene DNA hypomethylation [87,88] and consequent genetic instability [150]. SAM counteracts global DNA hypomethylation [88] and inhibits the development of preneoplastic foci in rat liver [88,89,90,91,92]. The hypomethylating agent, 5-azacytidine, prevents this SAM effect [151].

The changes [38]. The nuclear localization is correlated with the histone H3K27 tri-methylation. MATI/III activity supplies of MATs expression in HCC may also interfere with the protein methylation. Conformational signals in the C-terminal domain of MATI/III are responsible for its nucleo-cytoplasmic distribution the SAM necessary for this epigenetic modification. The MATII isozyme (constituted by MATα2 and MATβ2 proteins) provides SAM locally by interacting with chromatin-related proteins implicated in histone modification, chromatin remodeling, transcription regulation, and nucleo-cytoplasmic transport [40]. MATα2 and MATβ2 interact with the MAF oncoproteins, MAFK (v-MAF avian musculoaponeurotic fibrosarcoma oncogene family, protein K) [40]. The latter is a transcription activator or repressor that forms heterodimers with MAF recognition elements of DNA [152]. MATα2 and MATβ2 are recruited specifically to MafK target genes and are required for their repression by MafK and its partner, Bach1. Because the catalytic activity of MATIIα is required for the MafK target gene repression, MATIIα could provide SAM locally on chromatin [152].

## 7. Therapeutic Effect of SAM in Liver Disease

The decrease in *MAT1A* gene expression with a consequent reduction of the MATI and MATIII isoenzymes leads to a marked fall of SAM liver content during acute and chronic liver disease, which predisposes the subject to the development of liver cancer [57,153,154]. This has been well reproduced experimentally by the MAT1A-KO mice model of Mato and coworkers [59], which allowed researchers to typify the effects of the *MAT1A* fall in hepatocarcinogenesis. These mice exhibited hepatomegaly in the absence of histologic abnormalities at three months of age, mononuclear infiltration in portal areas and macrovesicular steatosis of 25–50% of liver parenchyma at eight months, and HCC development at 18 months [59]. These changes were associated with the expression of many acute phase-response and inflammatory markers and overexpression of growth-related genes, including early growth response 1 and proliferating cell nuclear antigen. At three months, knockout mice were more susceptible to the induction by a choline-deficient diet of fatty liver and lipogenesis and impairment of very low density lipoprotein (VLDL) assembly with a decrease of triglyceride excretion; these changes were corrected by SAM administration [60].

The association of liver injury with the decrease in SAM synthesis suggests a possible therapy based on the restoration of normal SAM levels. It was observed that the expression of *SREBP-1*, which activates genes involved in the synthesis and trafficking of cholesterol and other lipids, and the lipogenesis increased when the cellular methylation (critical for the synthesis of phosphatidylcholine) was limited [155]. Decreased SAM synthesis causes elevated SREBP-1-dependent transcription and lipid droplet accumulation [155]. During alcoholic steatohepatitis, there occurs ROS generation by the cytochrome P450 and translocation of bacteria from the gut, with a consequent release of TNFα and pro-inflammatory cytokines by Kupffer cells and immune system activation [155]. Furthermore, the malnutrition of these patients causes vitamins B1, B6, B12, and folate deficiency. Vitamins B6 and B12 are required for the activity of methionine synthetase, betaine homocysteine methyltransferase, and cystathionine synthetase (Figure 1), and, thus, for the synthesis of SAM and GSH [156]. Therefore, vitamin B6 and B12 deficiency would impede methylation reactions and would increase peroxidative damage.

SAM treatment largely prevents ethanol toxicity, fat accumulation, and liver damage in rats [72,74], mice [73] and baboons [75]. This effect has been at least partially explained by the capacity of SAM to maintain an elevated GSH pool and low acetaldehyde concentration [72,157]. A study on the effect of SAM plus dilinoleoylphosphatidylcholine (DLPC) treatment on lipid composition and acute ethanol toxicity in isolated perfused liver showed that this treatment enriches liver membranes of polyunsaturated phosphatylcholine molecular species and maintains normal levels of mitochondrial GSH and oxygen consumption by liver cells [158].

Different clinical trials of SAM in alcoholic liver disease have indicated that this treatment induces an increase of plasma and/or hepatic GSH levels [82,83]. A two year Spanish multi-center study evaluated the effects of oral SAM in 123 cirrhotic patients [80]. Sixty-two of these patients (53 males, nine females) were treated with 1.2 g/day of SAM, and 61 patients received placebo. All causes of mortality, including liver transplantation, complications of liver disease, and clinical biochemistry, were included in this study. It was found that a combined all-cause mortality/transplantation end point fell from 30% in the placebo group patients to 16% in SAM-treated patients, but the difference between these effects was not statistically significant. The significance was reached when the patients with more advanced disease were excluded from the analysis.

An update of the 2001 systematic review by members of the Cochrane Collaboration identified nine randomized control trials that met their inclusion criteria [159]. These studies, including the trial discussed above [80] that was the only one considered to have used adequate methodology, recruited a total of 434 patients [159]. However, in this controlled study considering the trials made between 1950 and 2006, no evidence was found to either support or refute the use of SAM for patients with alcoholic liver diseases [159].

The chemoprevention of human HCC by SAM was also the subject of different studies. SAM reduces HCV expression in human Huh7 HCC cells, and it was found that this effect involves the activity of modulatory antioxidant enzymes and the restoration of GSH biosynthesis and MAT1A/MAT2A turnover in HCV expressing cells [160]. Furthermore, recent research showed that the treatment of chronic hepatitis C with SAM, betaine, and pegIFNα/ribavirin improves the early virological response that may be considered a precancerous condition [161], restore STAT1 methylation, and improve IFN signaling in cell lines harboring HCV [133]. Collectively, available data suggest that chemoprevention by SAM of HCC in patients with chronic hepatitis C is an achievable objective; however, it has not yet been reached [162]. Concerning the relationship between HBV infection and SAM, it has been observed that, in HCC samples, the levels of the X protein of HBV are correlated with MAT2B, and the X protein inhibits the apoptosis in HCC cells by enhancing the expression of *MAT2A* and decreasing the production of SAM [163]. Furthermore, SAM operates a protective effect on hepatic ischemia-reperfusion injury during hepatectomy in HCC patients with chronic HBV infection [164,165].

## 8. Possible Effects of the Manipulation of the MAT1A:MAT2A Switch

The decrease of MAT1A expression and the rise of MAT2A expression characterizes hepatocarcinogenesis, which may be modified through the miRNAs that influence the MAT1A:MAT2A ratio. Indeed, the induction of *MAT1A* expression in liver tumor Hep3B and HepG2 cell lines by the individual knockdown of miR-664, miR-485-3p, or miR-49, which are upregulated in HCC cells, inhibits the proliferation and induces apoptosis, while the combined knockdown exerts additional effects on the same parameters [68]. Furthermore, it has been shown that the subcutaneous and the intra-parenchymal injections of Hep3B cells stably overexpressing these three miRNAs promote tumorigenesis in mice [68].

*MAT2A* over-expression is higher in human HCCs with poor prognosis and in fast progressing liver tumors of rats genetically susceptible to HCC progression compared to the tumors developed in rats genetically resistant to hepatocarcinogenesis [65]. Additionally, *MAT2A* cross-talks with polyamine synthesis in colon and liver carcinoma [166], and the sumoylated Matα2 protein protects human colon and liver cancer cells from apoptosis by regulating BCL2 expression [167]. Furthermore, we recently demonstrated an oncogenic activity of both *MAT2A* and *MAT2B* genes [168]. These finding suggest that these genes could be targets of anticancer therapies.

Recent research indeed showed that miR-21-3p lessens *MAT2A* and *MAT2B* expression in HepG2 cells by targeting their 3’-primer untraslated regions (3′-UTRs); it also inhibits cell growth [169]. Furthermore, we found that miR-203 expression is inversely correlated with *MAT2A* and *MAT2B* expression and the expression of HCC proliferation and aggressiveness markers [168]. MiR-203 expression is genetically regulated and contributes to determining patients’ outcomes [168]. MiR-203 transfection of HepG2 and Huh7 liver cancer cells targets the 3′-UTR of *MAT2A* and *MAT2B* and strongly inhibits the expression of *MAT2A* and *MAT2B* mRNAs and MATα2 and MATβ2 proteins. It also induces an increase in SAM content, inhibits cell growth, cell migration, and invasiveness, suppresses the expression of stemness markers, and induces apoptosis [168]. These findings suggest that miR-203 expression could predict HCC prognosis and function as a biomarker for patient stratification, drug selection, and efficacy, which underlines the need for further work to evaluate the therapeutic potential of miR-203 mimics against HCC.

## 9. Conclusions

Accumulating evidence indicates that the alterations of the methionine cycle and the connected folate and polyamine cycles play a fundamental pathogenetic role in liver cancer. The early demonstration of a SAM fall and a MAT1A/MAT2A switch during liver injury and hepatocarcinogenesis, the generation and the analysis of MAT1A-KO mice, and the demonstration of the anticancer effect of SAM treatment represent some milestones for the elucidation of the pathogenesis and the development of new therapies for liver cancer.

Researchers from different laboratories contributed to the discovery of multiple mechanisms of the SAM anticancer effect involving DNA and protein methylation, DNA stability, signal transduction, and glycolytic metabolism. Studies of rats differently susceptible to hepatocarcinogenesis have shown that most of these mechanisms are under genetic control. The observation of significantly stronger alterations of the methionine cycle in human HCCs with poorer prognosis [67] suggests the existence of a genetic control of the alterations of the methionine cycle in human HCCs. This possibility is reinforced by the discovery—through comparative functional genomic analysis—of the existence of an evolutionarily conserved gene expression signature that discriminates HCC phenotypes able to progress differently in both rats and humans [93]. Therefore, the rat model comparatively analyzing rats differently susceptible to liver carcinogenesis may help to identify prognostic subgroups of human HCC and novel putative prognostic markers.

The chemoprevention by SAM of precancerous and cancerous experimental lesions has been well proven. However, no definitive demonstration is available of a curative effect of SAM for human preneoplastic and neoplastic liver lesions, especially for the advanced ones. In contrast, the pathogenetic role of MAT1A/MAT2A switch for hepatocarcinogenesis is well established. This strongly suggests the possibility that the correction of the early stages of liver cancer development has positive therapeutic effects. Recent results propose that some miRNAs could be used to this aim. However, the approach to correcting the alterations of the methionine and the folate cycles is at its beginning, and new research is needed to confirm and extend the present, promising early results.
